# Selective Cytogenetic Responses to Nano-Fertilizer Co-Exposure in *Allium cepa* L.: Implications for Sublethal Phytotoxicity in Agroecosystems

**DOI:** 10.3390/jox16030071

**Published:** 2026-04-24

**Authors:** Olivia Torres-Bugarín, Alejandro Sánchez-González, María Luisa Ramos-Ibarra, Idalia Yazmín Castañeda-Yslas, Nina Bogdanchikova, Alexey Pestryakov, María Evarista Arellano-García

**Affiliations:** 1 Medicina Clínica de Especialidad, Decanato Facultad de Medicina, Universidad Autónoma de Guadalajara, Zapopan 45129, Jalisco, Mexico; 2 Facultad de Ciencias, Universidad Autónoma de Baja California, Ensenada 22860, Baja California, Mexico; alejandrosg@uabc.edu.mx (A.S.-G.); evarista.arellano@uabc.edu.mx (M.E.A.-G.); 3 Laboratorio de Toxicología Genética, Departamento de Salud Pública, División de Ciencias Veterinarias, Centro Universitario de Ciencias Biológicas y Agropecuarias, Universidad de Guadalajara, Guadalajara 44340, Jalisco, Mexico; luisa.ramos@academicos.udg.mx; 4 Centro de Nanociencias y Nanotecnología, Universidad Nacional Autónoma de México, Ensenada 22860, Baja California, Mexico; icastaneda@ens.cnyn.unam.mx (I.Y.C.-Y.); nina@ens.cnyn.unam.mx (N.B.); 5 Research School of Chemistry and Applied Biomedical Sciences, Tomsk Polytechnic University, 634050 Tomsk, Russia; pestryakov2005@yandex.ru

**Keywords:** nanoparticle–fertilizer interaction, cytogenetic biomarkers, sublethal stress, hormetic response, root meristem, *Allium* test

## Abstract

The intensive use of agricultural inputs and the increasing incorporation of nano-materials into crop management practices raise concerns about their ecotoxicological interactions in plant systems. This study evaluated phytotoxicity, cytotoxicity, and genotoxicity in *Allium cepa* L. under experimental nano-agrochemical exposure scenarios combining two conventional nitrogen fertilizers—ammonium sulfate (AS) and urea—with silver nanoparticles (AgNPs). Biological responses were assessed across fertilizer concentrations (0.03–0.5 g/L), applied individually, simultaneously, and sequentially, to identify modulatory effects of AgNPs on plant proliferative activity and genomic stability. Results showed the relative stability of morphophysiological indicators associated with root growth, whereas cytogenetic biomarkers exhibited selective alterations under specific conditions. Significant increases in genetic damage markers were detected at intermediate ammonium sulfate concentrations, suggesting sublethal phytotoxicity windows not reflected by macroscopic growth parameters. In addition, modulation of the mitotic index and absence of generalized genotoxic effects in most combined or sequential treatments indicate that AgNPs primarily acted as modulators of proliferative responses rather than direct cytotoxic agents. Overall, these findings highlight the dynamic and non-linear nature of nano-agrochemical interactions in plant systems and underscore the importance of multibiomarker approaches for the early detection of genomic instability. The results provide experimental evidence relevant to the environmental risk assessment of nano-enabled fertilization strategies under realistic mixed-exposure scenarios. This study contributes to advancing the ecotoxicological understanding of emerging agricultural technologies and supports the need for further mechanistic research and field-based evaluations to guide the safe and sustainable use of nanomaterials in crop production.

## 1. Introduction

The intensive use of agricultural inputs constitutes one of the main factors influencing the environmental quality and biological stability of modern agroecosystems [1,2]. Among these inputs, nitrogen fertilizers play an essential role in maintaining plant productivity [3,4]; However, their application under real conditions can generate complex physiological and genetic responses in non-target plants [2,5]. These responses become particularly relevant in contexts where multiple chemical stressors coexist, interact, and can unpredictably alter cellular processes and plant development.

In this scenario, the recent development of agricultural nanoinputs, including metallic nanoparticles, has expanded strategies to improve fertilization efficiency, phytosanitary control, and productive sustainability [6]. However, the increasing incorporation of nanomaterials into agricultural systems poses new challenges from an ecotoxicological perspective, particularly concerning their effect in complex mixtures and their potential to modulate conventional agrochemical-induced responses [7].

In parallel, advances in nanotechnology have promoted the incorporation of silver nanoparticles (AgNPs) into agriculture, primarily due to their antimicrobial properties that contribute to crop protection. Recent studies have shown that AgNPs can also influence cell division in both plants and mammals, stimulating cellular proliferation and growth [8]. This effect is attributed to their interaction with key processes such as DNA synthesis and cell cycle regulation [9]. Consequently, plants treated with appropriate concentrations of AgNPs may enhance nutrient uptake and promote their development, suggesting the potential to reduce dependence on nitrogen fertilizers and thereby mitigate their environmental impact.

Despite their potential benefits, the coexistence of AgNPs and nitrogen fertilizers in agricultural soils presents significant challenges for sustainability. While AgNPs may alter soil microbiota and affect nutrient availability, their ability to stimulate plant growth suggests a promising strategy for reducing reliance on conventional fertilizers. Nevertheless, it is essential to advance research on optimal dosages, mechanisms of action, and long-term effects of these technologies to develop sustainable agricultural practices that maximize productive benefits while minimizing associated environmental risks.

On the other hand, several studies have shown that the biological response of plants to chemical stressors does not always follow linear patterns, nor is it dose-dependent [8,9]. On the contrary, non-monotonic responses can be observed, characterized by maximum effects at intermediate concentrations, accompanied by physiological adaptation, proliferative compensation, and activation of cellular defense mechanisms’ non-monotonic response phenomenon, known as hormesis [10]. These phenomena reflect the complexity of cell cycle regulation, redox balance dynamics, and interaction between nitrogen metabolism and genomic stability in meristematic tissues [8,11].

In this context, the *Allium cepa* L. bioassay has emerged as a robust plant-based tool for assessing cytotoxicity and environmental genotoxicity due to its high sensitivity in detecting mitotic alterations, micronucleus formation, and other chromosomal abnormalities [12,13]. In addition, this model allows for the integration of different levels of biological organization, from morphophysiological responses related to root growth to cytogenetic biomarkers associated with genomic stability, providing a comprehensive approach to studying the impact of emerging pollutants [14].

Despite growing evidence of the individual effects of nitrogen fertilizers and metal nanoparticles, there is still limited information on biological responses to combined or sequential exposures to these stressors [2,15,16]. Understanding the nature of these interactions is fundamental for risk assessment in real agroecosystems, where crops can be simultaneously exposed to multiple compounds with different mechanisms of action [17].

Therefore, the objective of this study was to evaluate phytotoxicity, cytotoxicity, and genotoxicity in roots of *Allium cepa* L. exposed to two conventional nitrogen fertilizers (ammonium sulfate and urea) and to silver nanoparticles, applied individually, simultaneously, and sequentially. For this, morphophysiological, proliferative, and cytogenetic responses were analyzed under different fertilizer concentrations (0.03, 0.06, 0.25, and 0.5 g/mL) to identify possible nano-interaction patterns, agrochemical non-monotonic responses, and sub-lethal effects relevant from an ecotoxicological perspective.

## 2. Materials and Methods

### 2.1. Preparation of Solutions

#### 2.1.1. *Silver Nanoparticles (AgNPs, Argovit TM)*

The silver nanoparticles used in this study correspond to the commercial formulation Argovit ^TM^, a stable aqueous suspension supplied by the Vector-Vita Science and Production Center (Novosibirsk, Russia). This formulation contains 1.2% (*w*/*w*) metallic silver stabilized with 18.8% (*w*/*w*) polyvinylpyrrolidone (PVP). According to the manufacturer’s specifications and complementary analyses, AgNPs exhibit predominantly spheroidal morphology, with a size range of 1–90 nm and an average diameter of 35 12 nm, determined by transmission electron microscopy (TEM; JEOL JEM-2010, Tokyo, Japan). Silver content was confirmed by inductively coupled plasma optical emission spectrometry (ICP-OES; Varian, Palo Alto, CA, USA) before the start of experiments [18,19].

The physicochemical characterization in distilled water showed an average hydrodynamic diameter of 95 nm and a zeta potential of 14 mV, measured by dynamic light scattering with a Zetasizer Nano NS DTS-1060 (Malvern Panalytical Ltd., Worcestershire, UK). The UV–Vis absorption spectrum, recorded with an Agilent Cary 60 spectrophotometer (Agilent Technologies, Santa Clara, CA, USA), showed a peak of superficial plasmonic resonance at 424 nm, which confirms colloidal stability and adequate dispersion of nanoparticles [18,19].

#### 2.1.2. *Nitrogen Fertilizers*

Two fertilizers widely used in agriculture were urea (CO(NH_2_)_2_), a synthetic organic fertilizer with approximately 46% nitrogen, and ammonium sulphate ((NH_4_)_2_SO_4_), an inorganic fertilizer with 21% nitrogen and 24% sulfur [20]. Both compounds were dissolved in distilled water and applied at the following concentrations: 0.03, 0.063, 0.25, and 0.5 g/L. The concentration range was defined based on internationally recognized thresholds for nitrate in drinking water [21]. According to the World Health Organization (WHO), adverse health effects, such as infantile methemoglobinemia, have been associated with nitrate concentrations exceeding 45 mg/L, while typical drinking-water levels in most regions remain below 10 mg/L. Additionally, urea, a widely used nitrogen fertilizer, is rapidly transformed in the environment into ammonia and subsequently into nitrate through microbial processes, contributing to nitrogen loading in water systems. Based on these criteria, the experimental concentrations were established at 50% of the most restrictive reference value, thereby allowing the inclusion of environmentally realistic exposure scenarios. This approach facilitates the identification of potential sublethal effects under conditions relevant to agroecosystems [21].

#### 2.1.3. *Positive Control*

Colchicine (0.4 mg/mL) was used as a positive control for mitotic inhibition. The roots were exposed for 1 h to induce metaphase arrest.

### 2.2. Allium cepa *L. Bulbs*

Commercial bulbs of *Allium cepa* L. with diameters of 2–3 cm were used, purchased in a local market. The bulbs were washed under running water, and pre-existing roots were carefully removed without damaging the basal meristem. The leaves were trimmed to a uniform length of 15 cm. Subsequently, the bulbs were acclimatised for 24 h in distilled water (pH 7.0) at 20 °C.

After acclimatization, each bulb was placed individually into a 50 mL Falcon^TM^ conical tube containing 10 mL of the experimental solution (control or treatment). The solutions were renewed every 24 h for a total period of 72 h.

At the end of the exposure, the number of roots per bulb and the length of roots were recorded; subsequently, the selected roots were cut with a scalpel, and flakes were placed for cytogenetic analysis.

### 2.3. Experimental Design

Twenty-seven experimental groups were established, divided into nine treatment categories; each group consisted of five independent biological units (Table 1). Each biological unit consisted of a bulb of *Allium cepa* L., individually placed in a 15 mL Falcon tube containing the solution corresponding to the treatment.

Nitrogen fertilizers were evaluated at four concentrations (0.03, 0.063, 0.25, and 0.5 g/L), yielding 27 experimental groups, including individual, combined, and sequential treatments.

The experimental design contemplated simple, sequential exposures to nitrogen fertilizers and silver nanoparticles to simulate potential scenarios of nanoagrochemical co-exposure under realistic agricultural conditions. This approach made it possible to evaluate both the dose-dependent effects of each agent and possible interactions arising from the order of exposure, including cytotoxic, genotoxic, or cell growth-modulatory responses.

The organization of treatments and exposure times at 24, 48, and 72 h is presented in Table 1.

### 2.4. Measurement of Root Growth

At the end of the experimental period, the bulbs were carefully rinsed with distilled water and dried on absorbent paper to remove excess moisture. Subsequently, all the roots of each bulb were counted, and their length in millimeters was measured, considering the distance from the basal plate to the root apex.

The mean root length per bulb was used as a quantitative indicator of phytotoxicity and proliferative activity of the radicular meristem. A decrease in root growth was interpreted as evidence of mitotic inhibition or treatment-induced cell damage. In contrast, an increase in root elongation was considered a possible stimulatory response or a hormetic effect associated with exposure.

### 2.5. Fixing and Storage of Samples

The roots were fixed individually in Carnoy’s solution (methanol: acetic acid, 3:1) to preserve the structural and chromosomal integrity of meristematic tissues. Subsequently, the samples were kept in the same solution until cytological processing, ensuring proper preservation of the cellular material for microscopic analysis.

### 2.6. Slide Preparation

Three roots per bulb were selected and subjected to acid hydrolysis with HCl for 10 min to facilitate cell separation. Subsequently, the roots were stained with acetic orcein for 40 min to highlight nuclear and chromosomal structures.

After staining, cytological preparations were performed using the crushing (squash) technique in 45% acetic acid, which allowed adequate dispersion of meristematic cells for observation.

The microscopic evaluations were carried out using a Carl Zeiss Primo Star microscope (Carl Zeiss Microscopy GmbH, Jena, Germany) equipped with a 40× objective lens, which enabled analysis of mitotic activity and treatment-induced chromosomal alterations.

### 2.7. Mitotic Index and Genotoxicity

The mitotic index (MI) was calculated as the percentage of cells in division—including prophase (P), metaphase (M), anaphase (A), and telophase (T) phases—to the total number of observed cells, according to the following expression:

$$\%\;MI\;=\frac{\mathrm{Cells}\;\mathrm{in}\;\mathrm{division}\;(P\;+\;M\;+\;A\;+\;T)}{\mathrm{Total}\;\mathrm{of}\;\mathrm{cells}\;\mathrm{observed}}\times 100$$

For each treatment, at least 1000 meristematic cells were evaluated in order to ensure the representativeness and reliability of the cytogenetic analysis.

Genotoxicity was assessed by recording micronuclei and chromosomal abnormalities. The micronuclei were analyzed as an independent biomarker. Total chromosomal abnormalities were calculated as the sum of lagging chromosomes, anaphasic bridges, sticky chromatin, C-mitosis, and polar deviations.

This approach allowed for an integrated assessment of proliferative activity and genomic stability, providing simultaneous evidence of cytotoxic and genotoxic effects induced by individual and combined treatments.

### 2.8. Statistical Analysis

All experiments were performed in triplicate using independent biological replicates (three bulbs per treatment), and the results are expressed as mean standard deviation (SD). Before inferential analysis, raw data were evaluated for distribution and homogeneity of variance. Since some variables showed a tendency toward Poisson-like distributions, mathematical transformations were applied to stabilize the variance and meet the assumptions of parametric analysis [22].

The number of roots, frequency of micronuclei, and total chromosomal abnormalities were transformed by the square root function (√x + 0.5). The root length was transformed using the log (x + 1) function. In the case of the mitotic index, as it is a proportional variable, the arcsine transformation of the square root (Arcsin √x) was used to improve normality and homogeneity of variances [23].

After verifying the statistical assumptions in the transformed data, a one-way analysis of variance (ANOVA) was performed. When statistically significant differences between treatments were detected, the Tukey multiple comparison test was applied to identify differences between averages. All statistical analyses and figure preparation were performed using GraphPad Prism version 10 (GraphPad Software, San Diego, CA, USA). The statistical significance level was set to α = 0.05.

## 3. Results

This study evaluated the cytotoxic and genotoxic effects of different concentrations of urea and ammonium sulfate (ranging from 0.5 g/L to 0.03 g/L), applied individually or in combination with silver nanoparticles (AgNPs, Argovit™), using the Allium cepa model. The biomarkers analyzed included root number and length, the mitotic index (determined by the frequency of cells in each mitotic phase: prophase, metaphase, anaphase, and telophase), as well as the frequency of micronuclei and other chromosomal aberrations.

### 3.1. Morphophysiological Response of Root Growth

**Figure 1:**
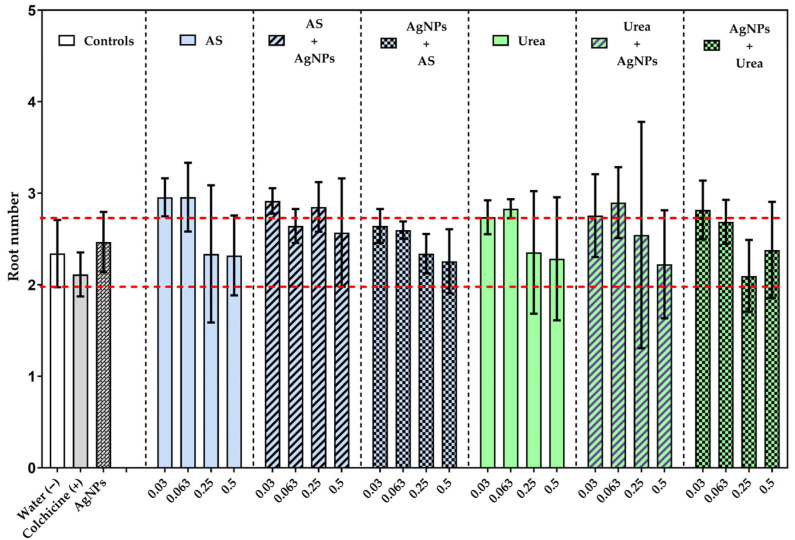
Average number of roots per bulb of *Allium cepa* L. exposed to individual and sequential treatments with ammonium sulfate (AS), urea, and silver nanoparticles (AgNPs). The bars are the mean and standard deviation. The red dotted horizontal lines indicate the negative control range (distilled water). Statistical differences between treatments were evaluated using ANOVA, followed by Tukey’s test [All dose groups 0.03/0.063 g/L vs. colchicine/AgNps]. The detailed results of multiple comparisons are presented in Supplementary Table S1 (Sheet 1).

The average number of roots per bulb in *Allium cepa* L. did not differ significantly among the evaluated treatments and the negative control (Figure 1). This outcome was kept in both individual exposures to nitrogen fertilizers and silver nanoparticles, as well as in combined and sequential treatments, regardless of the applied concentration. The observed stability suggests that root initiation constitutes a relatively conservative morphophysiological process, not sensitive to the evaluated nanoagrochemical co-exposure conditions.

**Figure 2:**
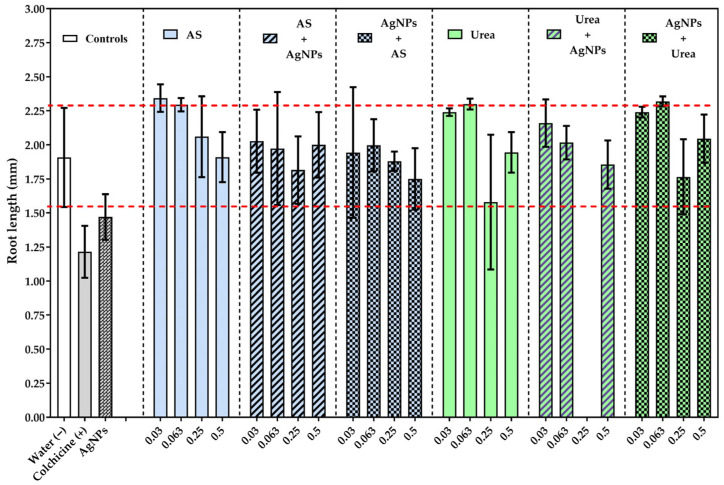
Average root length (mm) of *Allium cepa* L. exposed to nitrogen fertilizers ammonium sulfate (AS), urea, and silver nanoparticles (AgNPs) in individual and sequential treatments. The bars are the mean and standard deviation. The red dotted lines correspond to the range of variations in the negative control. Statistical analysis was performed using ANOVA and the Tukey test. [All dose groups 0.03/0.063 g/L vs. Colchicine/AgNps]. The complete multiple comparisons are shown in Supplementary Table S1 (Sheet 2).

Correspondingly, the mean root length also did not show significant growth inhibition relative to the negative control (Figure 2), indicating that root elongation remained functionally stable under most experimental conditions. However, some treatments at low concentrations showed significant differences compared to the positive control (colchicine) and to the treatment alone with silver nanoparticles *p* < 0.001, All dose groups 0.03/0.063 g/L vs. colchicine/AgNps], which evidence a differential cytotoxic response gradient among the agents evaluated.

In contrast, the colchicine-treated group (positive control) exhibited a marked reduction in root length, consistent with its well-known role as a mitotic inhibitor [24]. Similarly, the group exposed exclusively to AgNPs showed a significant decrease in root growth, although less pronounced than that observed with colchicine, suggesting partial interference with cell proliferation or elongation. Overall, the statistically significant difference (*p* < 0.01) between the water-treated group and the remaining experimental groups reinforces the sensitivity and reliability of this model for detecting phytotoxic effects.

Taken together, these results indicate that the morphophysiological parameters of root growth have limited sensitivity in detecting early cytotoxic effects, suggesting that the effects of nitrogen fertilizers and silver nanoparticles may manifest preferentially at the cellular and cytogenetic levels rather than in visible morphological alterations.

### 3.2. Modulation of Proliferative Activity

**Figure 3:**
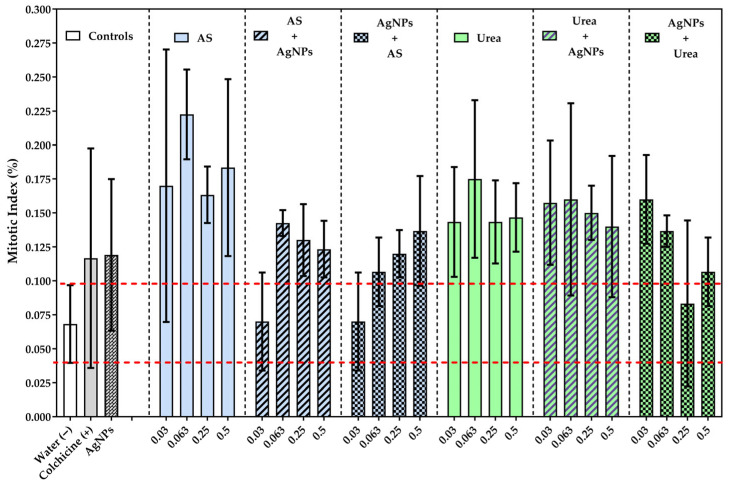
Mitotic index (%) in *Allium cepa* L. root meristematic cells exposed to ammonium sulfate (AS), urea, and silver nanoparticles (AgNPs) under different exposure schemes. The bars are the mean and standard deviation. The red dotted lines indicate the negative control interval. Differences between treatments were analyzed using ANOVA, followed by Tukey’s test. [AgNPs vs./AS 0.063 + AgNPs/Urea + AgNPs]. Detailed results are presented in the Supplementary Table S1 (Sheet 3).

The mitotic index showed a differential response between treatments (Figure 3). In particular, exposure to AgNPs significantly increased this parameter compared to the control group. Also, certain combinations with nitrogen fertilizers, at specific concentrations, reached significantly higher values than those observed with AgNPs alone. However, none of the combined treatments differed significantly from the negative control, suggesting that the modulation of proliferative activity was punctual and context-dependent, rather than indicative of widespread mitotic alteration.

Consistently, some sequential combinations with nitrogen fertilizers, particularly at specific concentrations, showed significantly higher mitotic index values than those observed with AgNPs alone, suggesting modulating effects associated with nanoagrochemical interactions [*p* < 0.001 AgNPs vs./AS 0.063 + AgNPs/Urea + AgNPs]. This pattern of response could indicate that the order and intensity of exposure influence the dynamics of meristematic cell proliferation.

However, in general, the combined treatments showed no significant differences compared with the negative control, indicating that the observed proliferative stimulation was selective and context-dependent rather than reflecting widespread mitotic disruption. Together, these results show that the mitotic index is a cytological biomarker with greater sensitivity, capable of detecting early responses to exposure to nitrogen fertilizers and silver nanoparticles.

### 3.3. Selective Micronucleus Induction

**Figure 4:**
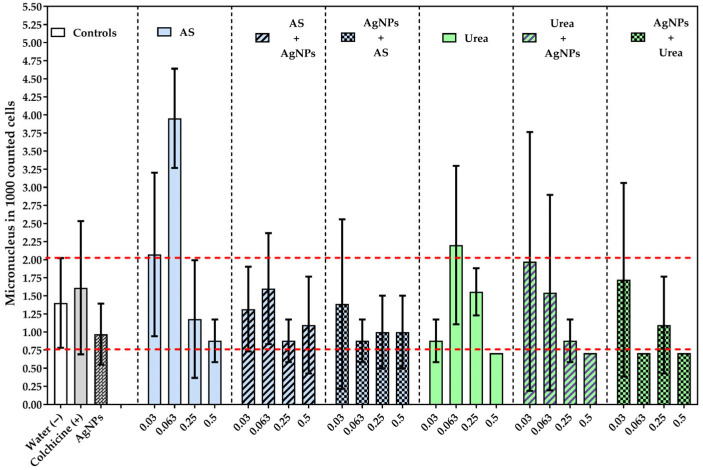
Frequency of micronuclei in meristematic cells of *Allium cepa* L. exposure to individual and sequential treatments with nitrogen fertilizers and silver nanoparticles. The bars are the mean and standard deviation. The dotted lines indicate the negative control’s range. Statistical analysis was performed using ANOVA and the Tukey test. [AS 0.063 vs. Water/Colchicine/AgNPs + SA 0.063/(AgNPs + AS 0.063]. The complete multiple comparisons are presented in Supplementary Table S1 (Sheet 4).

The frequency of micronuclei showed a differential pattern between treatments, characterized by a more focused than generalized response (Figure 4). The highest value was observed in the treatment with ammonium sulfate at a concentration of 0.063, which was significantly higher than the negative control, colchicine, and individually applied silver nanoparticles [*p* < 0.01 AS 0.063 vs. Water/Colchicine/AgNPs/[AS 0.063 +AgNPs/AgNPs + AS 0.063]. Also, this treatment differed from several combined and sequential AgNPs exposures, as well as from other concentrations of the same fertilizer, confirming that micronucleus induction depended largely on concentration and exposure context.

In contrast, most individual, combined, and sequential treatments showed no significant differences from the baseline control. Taking together, these results indicate that micronucleus formation did not respond to a uniform cytogenotoxic effect of co-exposure to nanoagrochemicals, but rather to a specific susceptibility condition primarily associated with ammonium sulfate at an intermediate concentration.

### 3.4. Total Chromosomal Abnormalities

**Figure 5:**
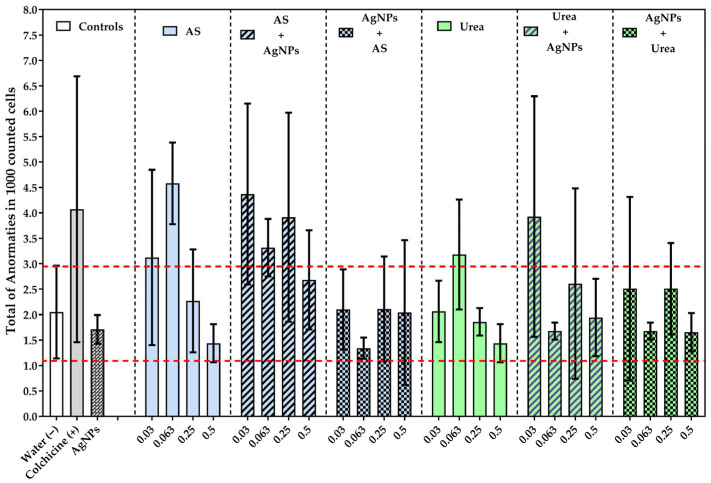
Total chromosome abnormalities (lagging chromosomes, anaphasic bridges, sticky chromatin, C-mitosis, and polar deviations) in root cells of *Allium cepa* L. exposed to nitrogen fertilizers and AgNPs. The bars are the mean and standard deviation. The dotted lines show the negative control interval. Statistical differences were evaluated using ANOVA, followed by Tukey’s test. [AS 0.063 vs. AgNPs/AS 0.03 + AgNPs vs. AgNPs]. The detailed results of multiple comparisons are presented in Supplementary Table S1 (Sheet 5).

Unlike the frequency of micronuclei, the total number of chromosomal abnormalities showed a less pronounced pattern, suggesting that not all treatments affecting genomic stability induced visible alterations in cell dynamics to the same extent (Figure 5). Although some treatments showed high mean values, most did not differ significantly from the negative control. The differences detected by the Tukey test were more restricted, highlighting the contrast between ammonium sulfate at 0.063 and AgNPs, as well as between sequential treatment of ammonium sulfate at 0.03 followed by AgNPs and AgNPs applied alone, [*p* < 0.01 AgNPs vs. AS 0.063/AgNPs vs. AS 0.03 + AgNPs].

This pattern suggests that, under the conditions evaluated, the set of chromosomal abnormalities was a more variable and less sensitive biomarker than the frequency of micronuclei for highlighting the genotoxic effect of treatments. Consequently, the overall genotoxic response appears to have depended not only on the presence of the agents evaluated but also on the set of alterations considered, indicating that exposure to treatments with the mixture of AgNPs and conventional fertilizers did not uniformly increase the sum of chromosomal damage indicators.

### 3.5. Synthesis of the Cytogenetic Pattern

Taken together, the cytogenetic damage biomarkers revealed a non-uniform response to the evaluated treatments. The frequency of micronuclei was the most sensitive indicator for detecting a genotoxic effect, particularly with ammonium sulfate at 0.063. In contrast, total abnormalities showed a more dispersed and statistically less consistent signal. This decoupling of biomarkers suggests that the interaction between nitrogen fertilizers and silver nanoparticles did not produce a generalized intensification of chromosome damage, but rather a selective modulation of specific cytogenetic events, depending on the concentration and sequence of exposure.

## 4. Discussion

### 4.1. Nitrogen Phytotoxicity and Differential Sensitivity of Biomarkers in Allium Strain

The results indicate that exposure to nitrogen fertilizers elicited biological responses that depended on both concentration and the evaluated biomarker. While the morphophysiological indicators associated with root growth—such as root number and root length—showed relative stability against most treatments, the cytogenetic biomarkers showed a greater sensitivity to detect alterations induced by these compounds. This pattern aligns with the recognized utility of the *Allium cepa* L. bioassay as an early-warning system for detecting sublethal cytotoxic and genotoxic effects, even in the absence of visible changes in plant development [25,26].

Phytotoxicity associated with nitrogen fertilizers, particularly in forms such as urea and ammonium sulfate, may involve mechanisms that transcend their nutritional function. It has been documented that these compounds can alter the osmotic and ionic balance of the root microenvironment, modify the medium pH, and induce oxidative stress, processes that may affect cell cycle dynamics and chromosomal stability [27,28]. In this context, the selective increase in micronucleate frequency observed at a specific concentration of ammonium sulfate suggests a critical window of genotoxic susceptibility, in which the physiological stress reaches levels sufficient to compromise genomic stability without yet causing a generalized inhibition of root growth.

This non-monotonic behavior reinforces the notion that plant response to agrochemical stressors does not necessarily follow linear dose–response patterns. Intermediate concentrations may induce more pronounced effects than extreme doses, possibly due to physiological adaptation, metabolic detoxification, or redistribution of nitrogen within plant tissue [13,29]. Such differential responses underscore the relevance of multibiomarker approaches in phytotoxicity studies, as early effects may manifest at the cytogenetic level before translating into detectable morphophysiological alterations.

### 4.2. Modulation of Proliferative Activity and Non-Linear Responses Associated with Silver Nanoparticles

Exposure to silver nanoparticles had specific effects on the proliferative activity of the root meristem, manifested as punctate increases in the mitotic index under certain experimental conditions. This type of response has been previously described in plant systems exposed to metallic nanoparticles. It is usually interpreted as an adaptive response or a hormetic phenomenon, depending on factors such as concentration, particle size, surface coating, and the bioavailability of metal ions [9,30].

Several studies have indicated that silver nanoparticle formulations stabilized with polyvinylpyrrolidone can promote plant growth without inducing significant cytogenetic damage in *Allium cepa* L., suggesting that the toxicity of these nanomaterials is not intrinsic or uniform, but highly contextual [8]. Consistent with this background, the variability observed in the present study suggests that AgNPs acted primarily as modulators of proliferative activity rather than as direct cytotoxic agents under the conditions evaluated.

This modulation could be associated with changes in redox signaling, cellular homeostasis, or activation of defense mechanisms, which coincides with the growing evidence for non-linear responses in plants exposed to nanomaterials [9]. In this sense, point stimulation of cell division could be interpreted as a compensatory response to moderate stress conditions rather than an indicator of uncontrolled proliferation.

Although it is well established that PVP itself does not induce cytotoxic or genotoxic effects, its presence as a stabilizing agent in the Argovit™ formulation should be considered when interpreting the observed responses. In particular, the potential contribution of PVP and the release of Ag^+^ ions may influence the biological effects attributed to AgNPs [8].

In this context, the inclusion of additional experimental groups containing PVP alone would be valuable to better understand its role and to further elucidate the interaction between the nanoformulation and nitrogen fertilizers. This approach would allow a more precise differentiation between nanoparticle-specific effects and those associated with other components of the formulation.

### 4.3. Nanoagrochemical Interaction: Implications for Genomic Stability and Ecotoxicological Assessment

One of the most relevant findings of this study was the absence of a generalized genotoxic effect in most combined or sequential treatments with nitrogen fertilizers and silver nanoparticles. This pattern suggests that the interaction between the two types of inputs does not necessarily follow simple additive or synergistic models, but depends on variables such as concentration, order of exposure, and the nature of the biomarker evaluated [26].

The observed responses can be interpreted within the framework of dynamic processes of physiological and cytogenetic modulation, in which co-exposure induces compensatory adjustments related to redox balance, the regulation of the mitotic cycle, and differential absorption of compounds in root tissue [31,32]. This type of non-linear interaction has been previously described in plant ecotoxicology and environmental nanotoxicology studies, where the bioavailability and toxicokinetics of agents can be substantially modified in multiple exposure scenarios [8,32,33].

The nanoagrochemical interaction should be understood as a dynamic phenomenon within the soil–plant system, in which factors such as the chemical speciation of nitrogen, nanoparticle aggregation, and metal ion release can modify the plant’s physiological and cytogenetic response [34]. Furthermore, it has been documented that nanoparticles can act both as oxidative stress inducers and as modulators of root growth and mitotic activity, depending on their concentration and the environmental context [8,30]. This functional duality makes it difficult to predict its effects on real agroecosystems characterized by the coexistence of multiple chemical stressors [35].

The results highlight the importance of integrating cytogenetic biomarkers in the ecotoxicological assessment of emerging agricultural technologies. The apparent stability of root growth against certain exposures could lead to an underestimation of risk if not considered as more sensitive indicators of genomic damage, such as micronucleus formation or chromosomal abnormalities [26,36]. In this sense, the assessment of interactions between conventional fertilizers and nanomaterials should be approached from a systemic approach that includes both morphophysiological responses as well as cellular and genomic alterations, considering that soil-soil interaction, plant-nanomaterial interactions can modify bioavailability and adaptive plant response [17,35].

Overall, the findings show that the response of *Allium* to co-exposure scenarios to nanoagrochemicals depends significantly on the evaluated biomarker, the applied concentration, and the order of exposure. The decoupling observed between stable morphological responses and selective cytogenetic alterations suggests that the potential effects of these interactions may initially manifest at the subcellular level before translating into visible changes in plant development [37]. Likewise, the punctate modulation of the mitotic index and the absence of a generalized increase in genetic damage indicate that silver nanoparticles acted as contextual modulators of proliferative activity rather than as universal inducers of cytotoxicity.

From an ecotoxicological perspective, these findings reinforce the need for multi-biomarker approaches to assess the impact of nanoinputs on agroecosystems, as mixed exposures can generate complex biological responses that are environment-dependent [17,38]. The integration of morphophysiological and cytogenetic indicators enables the prediction of sublethal and potentially cumulative effects, contributing to a more realistic risk assessment of the simultaneous use of traditional fertilizers and emerging agrochemicals.

Together, the results allow the identification of a differential response pattern in *Allium cepa* L. co-exposure scenarios to nanoagrochemistry, characterized by the relative stability of morphophysiological indicators and the greater sensitivity of cytogenetic biomarkers in detecting sublethal effects. This functional decoupling has been previously documented in plant bioassays, in which chromosomal alterations may manifest before visible changes in root growth are observed [26,36,38]. Likewise, the specific modulation of proliferative activity observed in this study suggests activation of adaptive mechanisms associated with cellular stress, a phenomenon described in plants exposed to metal nanoparticles and fertilizers under controlled experimental conditions [8,26].

Among the main limitations of this study is the use of a controlled experimental system based on the bioassay of *Allium strain*, which, although widely recognized as a sensitive tool for assessing environmental cytotoxicity and genotoxicity [26,36]. It does not fully reproduce the complexity of natural agroecosystems. Factors such as interaction with soil microbiota, adsorption of compounds in soil matrices, and variations in environmental conditions can modify the bioavailability and toxicity of fertilizers and nanoparticles [17]. In addition, the evaluation of a limited number of biomarkers restricts direct extrapolation of results to other crops or production systems.

In this context, it is suggested that future studies include multiple species and evaluations under conditions closer to the field in order to better understand the interaction between nitrogen fertilizers and nanomaterials. Likewise, the incorporation of molecular and physiological biomarkers would allow progress toward more comprehensive risk assessments and a more sustainable use of these technologies.

Taken together, the evidence suggests that nano-agrochemical co-exposure can reshape early genomic stability responses in crop plants, emphasizing the need for integrative multibiomarker approaches to support environmentally sustainable fertilization strategies and protect agroecosystem integrity.

## 5. Conclusions

The findings of this study support the hypothesis that the interaction between nitrogen fertilizers and silver nanoparticles modulates cytotoxic and genotoxic responses in Allium cepa in a dose- and context-dependent manner. The responses observed were non-linear and influenced by both concentration and exposure sequence, revealing sublethal phytotoxicity that would not be detected through morphophysiological parameters alone.

While root growth indicators remained relatively stable, cytogenetic biomarkers showed selective sensitivity under specific conditions, particularly at intermediate ammonium sulfate concentrations, where increases in genetic damage were detected. In contrast, the absence of generalized genotoxic effects and the modulation of the mitotic index across most treatments suggest that silver nanoparticles primarily acted as context-dependent modulators of proliferative activity rather than as direct cytotoxic agents.

These results highlight the importance of integrating cytogenetic biomarkers into ecotoxicological assessments, as reliance solely on macroscopic parameters may underestimate early or latent effects. Moreover, the lack of uniform responses across treatments indicates that nano-agrochemical interactions operate as dynamic processes governed by exposure conditions rather than producing consistent outcomes.

Overall, this study contributes to a better understanding of nano-enabled agricultural inputs by demonstrating that dose, exposure sequence, and biomarker sensitivity are key factors shaping plant responses. These findings support the use of Allium cepa as a sensitive model for detecting sublethal effects and reinforce the need for multibiomarker approaches in environmental risk assessment.

Future research should focus on elucidating the mechanistic basis of these interactions and on validating these findings under semi-controlled and field conditions to improve risk assessment frameworks and promote the safe and sustainable use of nanomaterials in agriculture.

## Figures and Tables

**Table 1:** Experimental design of individual, combined, and sequential treatments with nitrogen fertilizers and silver nanoparticles in *Allium cepa* L.

Experimental Group	Treatment Category	Dose	Exposure Time (Hours)		
			24	48	72
1	Water	-	Water	Water	Water
2	AgNPs Argovit^TM^	0.005 mg/mL	AgNPs	AgNPs	AgNPs
3	Colchicine	0.4 mg/mL	1 h	Water	Water
4–7 *	AS	*	AS	AS	AS
8–11 *	AS + AgNPs	*	AS	AgNPs	Water
12–15 *	AgNPs + AS	*	AgNPs	AS	Water
16–19 *	Urea	*	Urea	Urea	Urea
20–23 *	Urea + AgNPs	*	Urea	AgNPs	Water
24–27 *	AgNPs + Urea	*	AgNPs	Urea	Water

Urea (CO(NH_2_)_2_); AS-ammonium sulphate ((NH_4_)_2_SO_4_); * Fertilizer concentrations: 0.03, 0.063, 0.25, and 0.5 g/L. Each concentration was an independent experimental group.

## Data Availability

The original contributions presented in this study are included in the article/Supplementary Material. Further inquiries can be directed to the corresponding author.

## Supplementary Materials

The following supporting information can be downloaded at: https://www.mdpi.com/article/10.3390/jox16030071/s1. Table S1: Multiple-comparison analysis of mitotic index and chromosomal abnormalities.

## Abbreviations

The following abbreviations are used in this manuscript:

AgNPs	Silver nanoparticles
CO(NH_2_)_2_	Urea
(NH_4_)_2_SO_4_	Ammonium sulphate
MI	Mitotic index
